# Porous honeycomb structures formed from interconnected MnO_2_ sheets on CNT-coated substrates for flexible all-solid-state supercapacitors

**DOI:** 10.1038/srep18887

**Published:** 2016-01-04

**Authors:** Wen-Yin Ko, You-Feng Chen, Ke-Ming Lu, Kuan-Jiuh Lin

**Affiliations:** 1 Department of Chemistry, National Chung-Hsing University, Taichung (402), Taiwan

## Abstract

The use of lightweight and easily-fabricated MnO_2_/carbon nanotube (CNT)-based flexible networks as binder-free electrodes and a polyvinyl alcohol/H_2_SO_4_ electrolyte for the formation of stretchable solid-state supercapacitors was examined. The active electrodes were fabricated from 3D honeycomb porous MnO_2_ assembled from cross-walled and interconnected sheet-architectural MnO_2_ on CNT-based plastic substrates (denoted as honeycomb MnO_2_/CNT textiles).These substrates were fabricated through a simple two-step procedure involving the coating of multi-walled carbon nanotubes (MWCNTs) onto commercial textiles by a dipping-drying process and subsequent electrodeposition of the interconnected MnO_2_ sheets onto the MWCNT-coated textile. With such unique MnO_2_ architectures integrated onto CNT flexible films, good performance was achieved with a specific capacitance of 324 F/g at 0.5 A/g. A maximum energy density of 7.2 Wh/kg and a power density as high as 3.3 kW/kg were exhibited by the honeycomb MnO_2_/CNT network device, which is comparable to the performance of other carbon-based and metal oxide/carbon-based solid-state supercapacitor devices. Specifically, the long-term cycling stability of this material is excellent, with almost no loss of its initial capacitance and good Coulombic efficiency of 82% after 5000 cycles. These impressive results identify these materials as a promising candidate for use in environmentally friendly, low-cost, and high-performance flexible energy-storage devices.

Owing to the increased presence of portable, flexible and wearable electronic devices in daily life, a significant amount of research effort has been focused on developing lightweight, flexible and highly efficient energy storage devices^1^,^2^,^3^. Flexible supercapacitors feature safer operating conditions, a lower environmental impact, higher charge/discharge rate capabilities, energy densities higher than those of conventional capacitors, and higher power densities and longer life cycles than lithium ion batteries; for all of these reasons they have been considered as an important subject within the field of electrical energy storage^4^,^5^. Two-dimensional (2D) birnessite-type MnO_2_ sheets are one of the more promising candidates for active materials for use in such devices, as they are cost-effective, have naturally abundant raw materials, have excellent theoretical capacity and are non-toxic. They also feature several intriguing physicochemical properties, such as their large specific surface area as well as large interlayer distances (~7 Å) for facilitating proton and cation intercalations into the 2D layer structure, more so than other MnO_2_ structures^6^,^7^. However, irreversible agglomeration and parallel restacking of these sheets is a common occurrence that reduces the active surface area and limits electrolyte diffusion into the material, which in turn lowers the capacitance performance making it unsuitable for practical applications. Recently, the development of a three-dimensional (3D) MnO_2_ porous network assembled from interconnected 2D sheets has been proposed^7^,^8^,^9^, because this unique architecture not only can prevent sheet restacking but can also enhance the ion diffusion behavior of the 3D porous structure. For example, a one-step electrodeposition followed by low-temperature thermal annealing was recently used to directly grow mesoporous MnO_2_ sheets on Ni foam for pseudocapacitors which achieved a specific capacitance of ~201 F/g at 1 A/g^8^. In addition, MnO_2_ sheets interconnected to each other to form honeycomb pores were successfully prepared from electrospun carbon nanofibers reacted with a Mn precursor, and exhibited an excellent energy density of 41.1 Wh/kg at a power density of 3.3 kW/kg^9^. Although the excellent capacitance performance of such unique MnO_2_ architectures has been demonstrated, most of the studies were carried out using liquid electrolytes, which inherently feature leakage and integrity problems, leading to their incompatibility with flexible devices. As such, more work is required to determine the viability of these MnO_2_ materials for use as supercapacitors.

In this work, a 3D honeycomb MnO_2_ porous network consisting of interconnected MnO_2_ sheets on carbon nanotube (CNT)-based plastic substrates (denoted as honeycomb MnO_2_/CNT textiles) was successfully synthesized through a simple dipping-drying process followed by an electrodeposition process; well-dispersed multi-walled carbon nanotubes (MWCNTs) were coated onto commercial textiles using the dipping-drying process, which then served as a conductive framework for the subsequent electrodeposition of the MnO_2_ sheets. A stretchable solid-state supercapacitor was then assembled using honeycomb MnO_2_/CNT networks as a binder-free electrode and polyvinyl alcohol/H_2_SO_4_ as an electrolyte. Furthermore, only minor capacitance changes were observed in the supercapacitor when tested under bending conditions at various bending angles before and after 500 bending cycles, indicating its high mechanical robustness. This mechanical flexibility demonstrated the device’s potential for use in practical applications for flexible energy storage systems.

## Results

**Figure 1:**
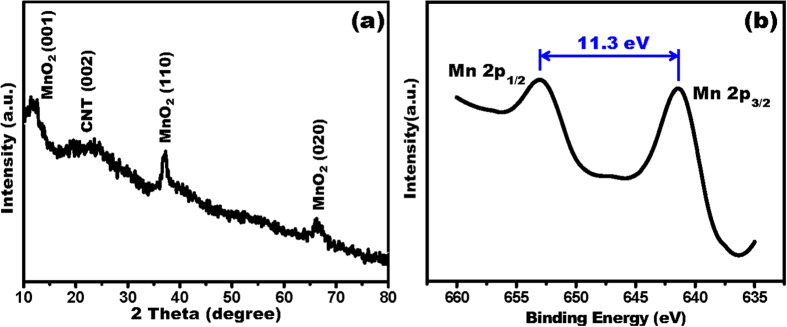
(**a**) X-ray diffraction pattern and (**b**) typical Mn2p X-ray photoelectron spectrum of the obtained product.

XRD measurements were used as a qualitative tool for phase identification. Figure 1a shows the XRD patterns (Cu Kα) of the synthesized MnO_2_/CNT composites. The broad diffraction band located at 2θ of 20–30° indicates the CNT materials^10^; the other three main peaks observed at 2θ values of 12.1, 37.2, and 66.6° correspond to the (001), (110), and (020) crystal phases of the 2D layered birnessite-type MnO_2_, which was indexed to a monoclinic type with space group C2/m and unit cell parameters of a = 5.150 Å, b = 2.844 Å, c = 7.159 Å, and β = 100.64° (JCPDS 42-1317)^11^. XPS measurements were also conducted to better understand the chemical composition of the as-synthesized deposited active materials (Fig. 1b). The high resolution Mn 2p spectrum shows the binding energies of the Mn 2p_3/2_ peak centered at 641.4 eV and the Mn 2p_1/2_ peak at 652.7 eV, with a spin-energy separation of 11.3 eV, which is in agreement with those previously reported for MnO_2_^12^. These results indicate the successful formation of layered birnessite-type MnO_2_.

**Figure 2:**
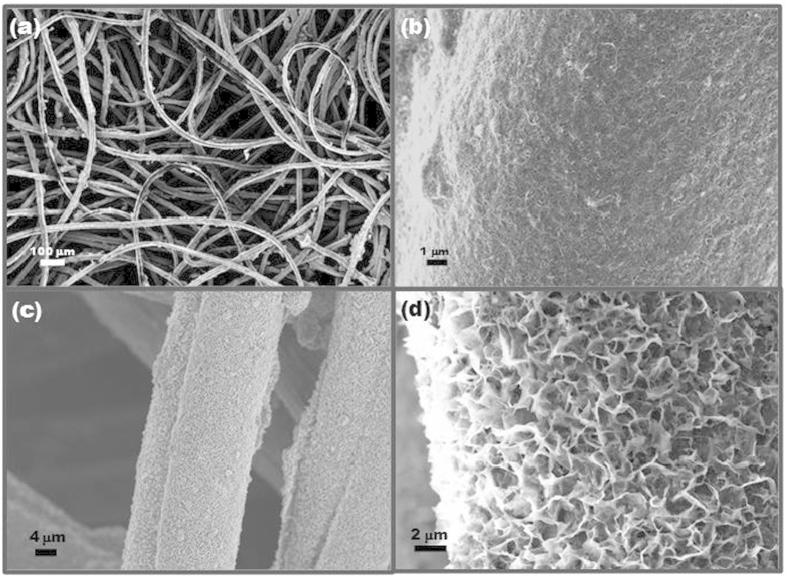
SEM images of (**a**) commercial textile, (**b**) MWCNT-coated textiles, and (**c**), (**d**) the prepared honeycomb MnO_2_/CNT 3D network textiles at different magnifications.

**Figure 3:**
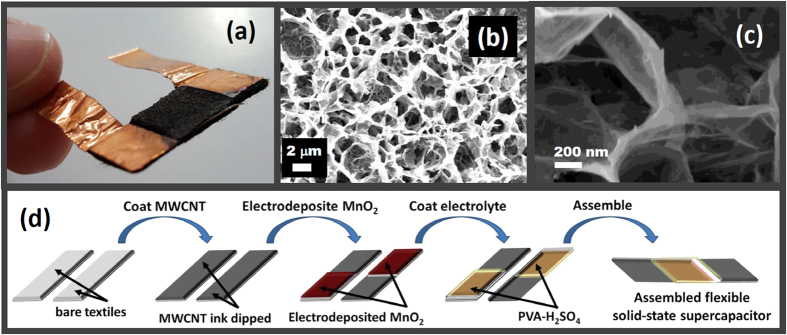
(**a**) Photograph of the flexible all-solid-state supercapacitor. (**b**,**c**) are the SEM images of the prepared honeycomb MnO_2_/CNT 3D networks. (**d**) Scheme of the fabrication process of the honeycomb MnO_2_/CNT textile-based flexible solid-state supercapacitors.

The morphology of the produced MnO_2_/CNT composites fabricated onto commercial textiles was characterized using SEM at different magnification levels, shown in Figs 2 and 3b,c. A typical microstructure of the pre-synthesized MWCNT-coated textiles is shown in Fig. 2b; high loading of the MWCNTs onto the commercial textiles can be observed, giving the film sufficient mechanical strength and electrical conductivity for further MnO_2_ deposition. Uniform MnO_2_ deposition on MWCNT-coated textiles, which consisted of sheets, was observed. These sheets are continuously interconnected and vertically orientated onto the surface of the MWCNT-coated textiles, constituting the 3D honeycomb-like pore walls (denoted as honeycomb MnO_2_/CNT textiles). Such 3D porous networks can minimize the restacking and aggregation of the MnO_2_ sheets, which enhances the electrolyte-accessible surface area and facilitates easy access of electrolyte ions into the interior of the electrodes^13^,^14^, which is a key factor in supercapacitor applications. The honeycomb MnO_2_/CNT textiles were directly applied as the binder-free electrodes for the fabrication of flexible all-solid-state supercapacitors with polyvinyl alcohol/H_2_SO_4_ as an electrolyte; the corresponding fabrication process is shown in Fig. 3d.

**Figure 4:**
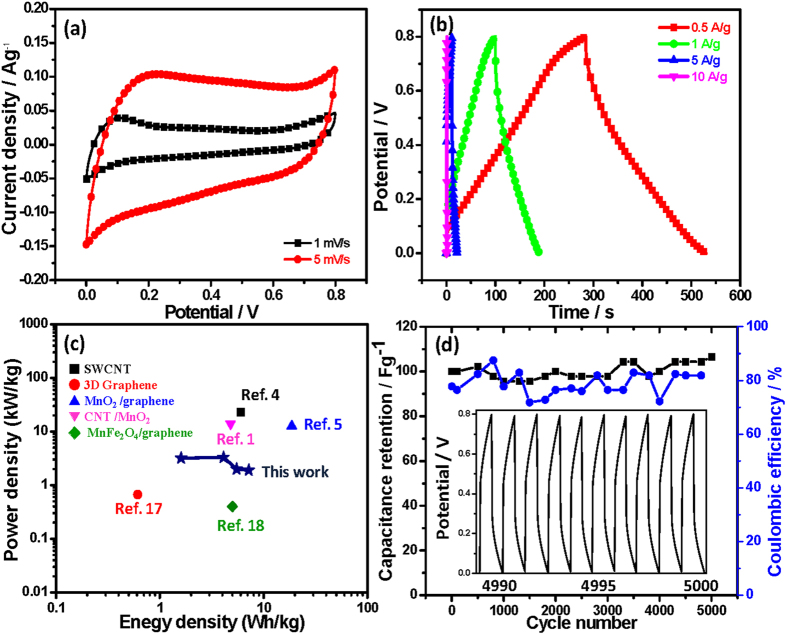
(**a**) CVs at different potential scan rates of 1 and 5 mV/s and (**b**) galvanostatic charge/discharge curves at different current densities of the flexible all-solid-state supercapacitors. (**c**) Performance comparison of various solid-state supercapacitors fabricated from carbon-based material electrodes and hybrid carbon-manganese oxide based electrodes by using PVA polymer relative electrolytes. The legend indicates the active electrode materials for each supercapacitor. (**d**) Capacitance retention (black) and Coulombic efficiency (blue) of the honeycomb MnO_2_/CNT textile device measured over 5000 charge/discharge cycles at a current density of 10 A/g. The galvanostatic charge/discharge curve for the device is shown in the inset.

CV measurements were performed to evaluate the electrochemical performance of the as-fabricated honeycomb MnO_2_/CNT textile-based supercapacitors. Figure 4a shows CV curves of the device in the 0 to 0.8 V range at scan rates of 1 and 5 mV/s. The shapes of the CV loops were found to be rectangular-like without obvious polarization curves and oxidation/reduction peaks, implying that diffusion of electrolyte ions, like protons, dominate the charging/discharging process. And the high power output ability of this double-layer capacitive behavior could be expected from the quick responses upon changing the scan direction. Extra energy storage was achieved by pseudocapacitive MnO_2_ which could undergo reversible redox reactions between Mn^4+^ and Mn^3+^ to induce the adsorption-desorption of protons inside MnO_2_ honeycomb structures. When voltage was applied to the MnO_2_/MnO_2_ supercapacitor, Mn^4+^ on the cathode reduced to III oxidation state, and protons combined with Mn^3+^ to form MnOOH. The combined double layer and pseudocapacitive behaviors contribute the total capacitance with high energy storage and high power out.

Galvanostatic CD curves of the as-prepared device were also collected at different current densities to evaluate the electrochemical properties and quantify the specific capacitance of the device, as shown in Fig. 4b. A small IR drop caused by equivalent series resistance was observed in the measurements, which includes electrode resistance, electrolyte resistance and contact resistance between the electrode and the electrolyte^15^. On the basis of the discharging curve line, the *C*_sp_ values of the working electrode were calculated to be 324, 248, 184, and 70 F/g at 0.5, 1, 5, and 10 A/g, respectively. The *E*_cell_ and *P*_cell_ values of our device were also calculated from the galvanostatic CDs (Table 1). A maximum energy density of 7.2 Wh/kg and a maximum power density of 3.3 kW/kg were achieved, values that compare favorably to those found in similar solid-state supercapacitor systems reported previously, systems that were also based on carbon-based material electrodes and hybrid carbon-manganese oxide based electrodes using PVA polymer-based electrolytes (see also the Ragone plot shown in Fig. 4c)^1^,^4^,^5^,^16^,^17^. The long-term cycling stability of the as-fabricated supercapacitor was further examined through a cyclic charge/discharge process at a constant current density of 10 A/g, which is shown in Fig. 4d. After over 5000 charging/discharging cycles, the supercapacitor device still remained at nearly the same initial capacitance before cycling, and the Coulombic efficiency held at ~82%, indicating remarkable long-term cycling stability of our honeycomb MnO_2_/CNT textile-based supercapacitors. The inset displayed no significant electrochemical change during the long-term charging and discharging process after 5000 cycles.

**Figure 5:**
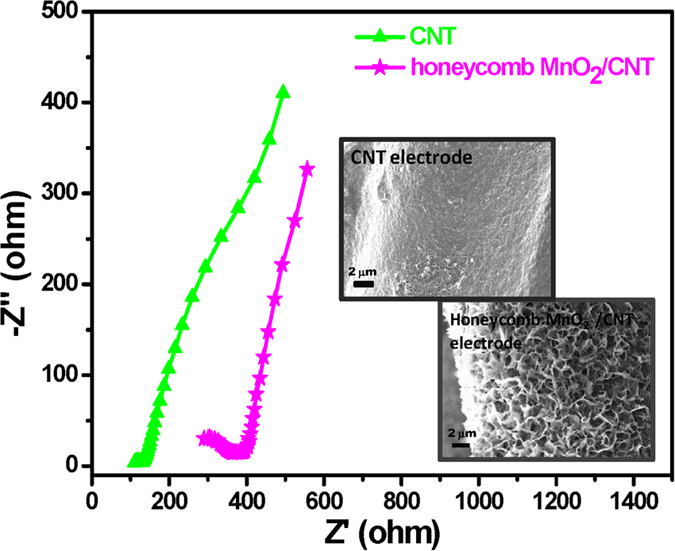
Nyquist impedance plots in the frequency range 0.1 Hz–200 kHz of the flexible solid-state supercapacitor device with CNT and honeycomb MnO_2_/CNT textiles, respectively.

In order to further study the electrochemical behavior, EIS measurements were carried out on CNT and honeycomb MnO_2_/CNT textile-based supercapacitors in a frequency range of 0.1 Hz to 200 kHz at open-circuit voltage, as shown in Fig. 5. A semicircle pattern is observed at a high frequency range while a linear Warburg-type line is observed at a low frequency range; the semicircle diameter corresponds to the charge transfer limiting process and the charge transfer resistance (*R*_ct_) at the surface and interfaces, and the Warburg plot is associated with the ion diffusion/transport from the electrolyte to the electrode surface^18^. It should be noted that for honeycomb MnO_2_/CNT textiles, a larger semicircles observed compared to that of the CNT-based device, demonstrating a higher interfacial charge-transfer resistance caused by the poor electrical conductivity of the MnO_2_ sample. However, a sharper Warburg plot for the honeycomb MnO_2_/CNT textiles compared to the CNT device indicates that the device possesses good capacitive behavior with rapid ion diffusion. These results demonstrate that the honeycomb MnO_2_/CNT textiles constructed from cross-walled and interconnected sheet-architectural MnO_2_ on CNT-based plastic substrates have the capability to enhance electrolyte interaction with the active materials and facilitate the penetration of electrolyte into the MnO_2_ surface, providing a better pathway for ion transport and resulting in competitive capacitive performance.

**Figure 6:**
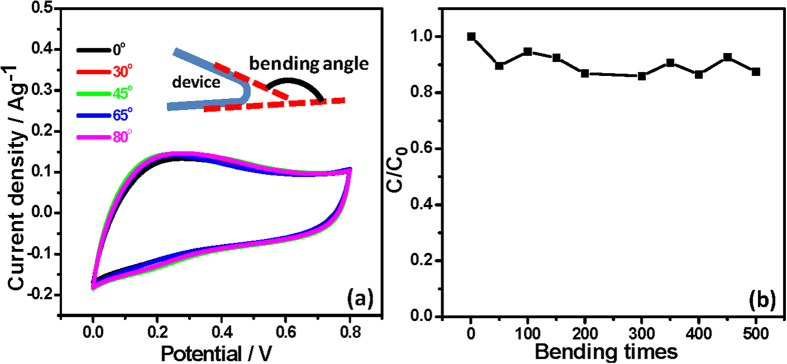
(**a**) CV curves of the honeycomb MnO_2_/CNT textile-based supercapacitor at a scan rate of 10 mV/s before and after bending with different bending angles. (**b**) Capacitance ratio (C/C_0_, where C_0_ is the initial capacitance) as a function of bending time (bending angle = 80°).

Lastly, we investigated the flexibility of the fabricated honeycomb MnO_2_/CNT textile-based supercapacitors. In order to evaluate the electrochemical performance for flexible energy storage, CV curves were obtained under different bending conditions. No significant current density decreases were observed as the bending angles changed from 0° to 80°, demonstrating that CNTs were strongly immobilized on the textiles, and the conducting networks were not disrupted after bending. The rectangle-shaped and nearly overlapping CV curves implied that the inner structures of the honeycomb MnO_2_ were well-maintained under these conditions (Fig. 6a). Noticeably, the honeycomb MnO_2_/CNT textiles are flexible and robust enough to tolerate long-term and repeated bending, and the *C*_sp_ value after 500 bending cycles was approximately 87% of the initial *C*_sp_ value (Fig. 6b). This clearly demonstrates that this honeycomb MnO_2_/CNT textile-based solid-state supercapacitor has robust flexibility and a long cycle life when used as a flexible energy storage device.

## Discussion

3D MnO_2_ frameworks constructed from 2D birnessite-type MnO_2_ sheets have been proven to be an appealing electrode material for flexible supercapacitors recently due to its capability to offer more electrolyte transport paths for the electrons transfer and protons/cations diffusion, which allows enhanced charge transport efficiency through the electrodes during charge/discharge process and leads to good supercapacitive performances. 3D honeycomb-like pore walls formed by MnO_2_ sheets can be observed (Figs 2 and 3). And the investigations of XRD and XPS confirm that the MnO_2_ sheets are birnessite-type crystal structure (Fig. 1). Moreover, the EIS data indicate that the based device possesses good capacitive behavior with rapid ion diffusion, demonstrating its capability to provide a better pathway for ion transport and resulting in better capacitive performance (Fig. 5).

In conclusion, we have created a 3D honeycomb porous MnO_2_/CNT supercapacitor, assembled from cross-walled and interconnected sheet-architectural MnO_2_ deposited onto CNT-based plastic substrates. With this unique architecture, the device shows high gravimetric specific capacitance of 324 F/g at 0.5 A/g, a comparable energy density of 7.2 Wh/kg and power density of 3.3 kW/kg, excellent cycling stability of ~100% capacity retention after 5000 cycles, and good mechanical flexibility, indicating the electrodes may have promising potential for high-performance, light-weight supercapacitors.

## Methods

### Materials

MWCNTs were produced as the procedure in our previous work. Sodium dodecyl sulfate (SDS, 98.5%) and manganese(II) sulfate monohydrate (MnSO_4_•H_2_O, 99%) were purchased from Sigma Aldrich. Sodium sulfate (Na_2_SO_4_, 99.0%) and polyvinyl alcohol (PVA, hydrolyzed 86–89%) were obtained from SHOWA and Alfa Aesar, respectively. All chemicals were used in the experiments without any further purification.

### Preparation of MWCNT ink

MWCNTs were produced using chemical vapor deposition method (First Nano’s EasyTube 2000 system) under a mixture of ethylene and methane with iron oxide powder as a catalyst. The MWCNTs were purified with concentrated hydrochloric acid, followed by washing with water, precipitation, and drying under vacuum, with subsequent heat treatment in air at 400 °C. To yield stable dispersions of MWCNTs as the ink for coating the textiles, brief sonication of 100mg MWCNT powder was carried out using a probe type sonicator (SONICS® VCX750, supplied by Sonics & Materials, Inc.) in 100mL aqueous solution containing 0.23% sodium dodecyl sulfate as a surfactant.

### Preparation of honeycomb MnO_2_/CNT textiles

Commercial textiles with high flexibility, low weight, good mechanical strength, and hierarchical surface morphology were used as the substrates for making the honeycomb MnO_2_/CNT networks. The preparation involved MWCNTs being coated onto commercial textiles using a dipping-drying process, with a subsequent controllable electrochemical deposition of the interconnected MnO_2_ sheets onto the as-prepared conductive MWCNT-coated textiles. First, a piece of commercial textile (1 × 2 cm; fabric store Taichung) was dipped into the MWCNT ink and immediately removed, followed by drying in a vacuum oven at 120 °C for 15 min, and then washing with excess water to rinse off the surfactants. The dipping-drying process was repeated five times, at which point the final conductive MWCNT-coated textile was obtained.

Electrochemical deposition synthesis of the interconnected MnO_2_ sheets was accomplished in an aqueous solution of 0.01 M Mn_2_SO_4_ and 0.1 M Na_2_SO_4_ at ambient temperatures using an Instruments 672A electrochemical system (CH Instruments) with a three electrode system, where the conductive MWCNT-coated textile acted as the working electrode, with a Ag/AgCl reference electrode (immersed in a 3 M KCl filling solution saturated with AgCl), and a Pt foil counter electrode. A constant potential of –1.8V was applied during the deposition process. After electrodeposition, the electrodes were rinsed with large amounts of Milli-Q water to remove any unreacted materials and excess electrolyte. The resulting MnO_2_/CNT composites, denoted as honeycomb MnO_2_/CNT networks, were dried overnight under normal atmosphere. The mass of the as-deposited MnO_2_ nanomaterial was measured using the weight difference of the sheet networks before and after coating onto the MWCNT-coated textiles.

### Fabrication of flexible solid-state supercapacitors

The H_2_SO_4_/PVA electrolyte was prepared by mixing 3 g of PVA and 3 mL of H_2_SO_4_ via magnetic stirring in 30 mL of Milli-Q water at a controlled temperature of 110 °C for 20 min. Two strips of the obtained honeycomb MnO_2_/CNT networks were immersed into the H_2_SO_4_/PVA electrolyte at 85−90 °C for 2 min, then removed and dried overnight under normal atmosphere. After evaporation of the water, the two film electrodes obtained were assembled together with a 200 μm thick filtration membrane (Grade No. 1, Toyo Roshi Kaisha, Ltd.) as a separator, and subsequently pressed together with polyethylene terephthalate films under a pressure of 1.5 kg/cm^2^ to form the final integrated device studied in this paper.

### Material characterization

The morphology of the obtained products was analyzed through a Zeiss Ultra-Plus field emission scanning electron microscope (FESEM) with an accelerating voltage of 3 kV. Powder X-ray diffraction (PXRD) was carried out on an analytical X’Pert Pro MRD equipped with Cu Kα as the X-ray source (λ = 0.15418 nm) at a scan rate of 0.033°/s in the 2θ range 10−80°. X-ray photoelectron spectroscopy (XPS) data were recorded using an ULVAC-PHI PHI 5000 VersaProbe X-ray photoelectron spectrometer with Al Kα X-ray beams as the excitation source.

### Electrochemical measurements

Cyclic voltammetry (CV), galvanostatic charge/discharge (CD), and electrochemical impedance spectroscopy (EIS) were applied to evaluate all the electrochemical properties of these materials. CV and galvanostatic CD measurements were recorded on an Instruments 672A electrochemical station (CH Instruments), where CV curves were recorded between 0 and 0.8 V at scan rates in the range of 1−10 mV/s, and the CD measurements were performed galvanostatically in the potential range 0−0.8 V at current densities in the range of 0.5~5 A/g. EIS measurements were conducted using a PARSTAT 2263 Advanced Electrochemical System in the frequency range of 0.1 Hz to 200 kHz at an AC amplitude of 10 mV. The specific capacitance (*C*_sp_) was calculated from the galvanostatic discharge curves using Eq. (1). In symmetric supercapacitors, the cell capacitance (*C*_cell_) is 1/4 of the specific capacitance of an electrode in a two-electrode configuration. The energy density (*E*_cell_) and power density (*P*_cell_) of a supercapacitor cell in the Ragone plots were calculated using Eq. (2) and (3).


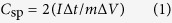



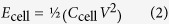



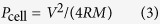


Where *I* is the discharge current, Δ*t* is the discharge time, *m* is the mass of active materials in one electrode, Δ*V* is the voltage change excluding the internal resistance (IR) drop, *V* is the applied potential, *M* is the total mass of active materials on both electrodes, and *R* is the internal resistance^19^,^20^.

## Additional Information

**How to cite this article**: Ko, W.-Y. *et al.* Porous honeycomb structures formed from interconnected MnO_2_ sheets on CNT-coated substrates for flexible all-solid-state supercapacitors. *Sci. Rep.* **6**, 18887; doi: 10.1038/srep18887 (2016).

## Figures and Tables

**Table 1:** Electrochemical properties of the honeycomb MnO_2_/CNT textile device.

Current density (A/g)	Specific capacitance (Csp) (F/g)	ESR	E_cell_ (Wh/kg)	P_cell_ (kW/kg)
0.5	324	433	7.2	1.9
1	248	400	5.5	2.0
5	184	240	4.1	3.3
10	70	251	1.6	3.2
